# Combination Treatment With Paclitaxel, Carboplatin, and Cetuximab (PCE) as First-Line Treatment in Patients With Recurrent and/or Metastatic Nasopharyngeal Carcinoma

**DOI:** 10.3389/fonc.2020.571304

**Published:** 2020-10-07

**Authors:** Yuri Ueda, Tomohiro Enokida, Susumu Okano, Takao Fujisawa, Kazue Ito, Makoto Tahara

**Affiliations:** Department of Head and Neck Medical Oncology, National Cancer Center Hospital East, Kashiwa, Japan

**Keywords:** nasopharyngeal cancer (NPC), PCE, paclitaxel, carboplatin, cetuximab

## Abstract

**Background:** Platinum-containing doublet chemotherapy regimens are generally considered the standard first-line systemic therapy for recurrent or metastatic (R/M) nasopharyngeal cancer (NPC). Gemcitabine (GEM) plus cisplatin (CDDP) has become a standard therapy based on a phase 3 study in several countries, yet this regimen sometimes affects quality of life due to nausea or appetite loss. Here, we present the manageable toxicity and promising activity of paclitaxel + carboplatin + cetuximab (PCE) therapy for R/M NPC.

**Materials and Methods:** We conducted a retrospective review of patients with R/M NPC who were treated with PCE from 2013 to 2019 at the National Cancer Center East, Kashiwa, Japan. PCE consisted of PTX 100 mg/m^2^ on days 1 and 8; CBDCA area under the blood concentration–time curve (AUC) 2.5 on days 1 and 8, repeated every 3 weeks; and cetuximab at an initial dose of 400 mg/m^2^, followed by 250 mg/m^2^ weekly, as reported in the paper.

**Results:** Fourteen patients were identified, consisting of 10 males and 4 females with a median age 59.6 years (range, 43–74). Among the 12 of 14 patients assessed for efficacy, overall response rate was 58.3%, with 2 complete responses and 5 partial responses. On median follow-up of 23.8 months, median overall survival was not reached with observed death events of 2. Median PFS was 4.1 months (95% CI, 2.6–5.6 months). Two patients experienced disease progression during cetuximab maintenance and restarted PCE treatment, then achieved partial response again. The most common grade 3 or 4 adverse events were neutropenia (21.4%) and skin reaction (14.3%). No treatment-related death was observed.

**Conclusion:** Although the number of study population was small, our results suggest that PCE is feasible and potentially effective for R/M NPC, with a 58.3% response rate and 4.1-month PFS. Further prospective evaluation is warranted.

## Introduction

Nasopharyngeal cancer (NPC) is a rare cancer in Japan but is common in several East Asian countries. Although the prognosis of NPC is basically favorable due to its high sensitivity to chemotherapy and radiotherapy, that of recurrent and/or metastatic (R/M) NPC is limited. A platinum-containing regimen is the standard therapy for R/M NPC, as with other head and neck squamous cell carcinoma (HNSCC). Although 5FU+cisplatin (CDDP) has long been the standard treatment (1), gemcitabine (GEM)+CDDP was shown in 2016 to provide better progression-free survival (PFS) and overall survival (OS) over 5FU+CDDP (2) and is now a standard therapy in several countries. However, this regimen sometimes affects quality of life due to nausea or appetite loss. Moreover, GEM has not been approved for the treatment of NPC in Japan. Meanwhile, following the EXTREME trial for R/M HNSCC, regimens containing cetuximab (Cmab)—an epidermal growth factor receptor (EGFR) inhibitor—are now a standard treatment for HNSCC (3). EGFR expression rate in NPC is reported to be about 80%, and the overexpression relates to inferior PFS and OS, leading to the expectation that Cmab will also show efficacy in NPC patients (4, 5). In fact, Cmab and other EGFR inhibitors were reported to be effective in locally advanced NPC, including Cmab+RT or Cmab/nimotuzumab+CRT (6–8). However, few studies have evaluated the efficacy and safety of chemotherapy plus Cmab for R/M NPC. A phase 2 study of Cmab+carboplatin (CBDCA) for heavily treated R/M NPC patients showed acceptable results with an overall response rate (ORR) of 11.7% and a disease control rate (DCR) of 60.0% (9). In 2019, a phase 2 study of nimotuzumab+5FU+CDDP as first-line treatment for R/M NPC showed similar OS and PFS as the EXTREME study (10). However, this regimen requires inpatient care or placement of a central access port for continuous intravenous infusion of 5-FU.

We previously conducted a phase 2 trial of the combination of paclitaxel (PTX), CBDCA and Cmab (PCE) for R/M HNSCC and showed promising clinical activity with acceptable toxicity. Toxicities were manageable in the outpatient clinic, with weekly adjustment of dosages according to toxicity (11). We then surmised that PCE would also be effective for R/M NPC and conducted a retrospective study. In addition, we attempted to evaluate the potential effect of PCE on the efficacy of subsequent treatment, herein with nivolumab.

## Materials and Methods

### Patient Selection

We retrospectively reviewed the case records of R/M NPC patients who were treated with PCE from 2013 to 2019 at the National Cancer Center East, Kashiwa, Japan. Inclusion criteria were as follows: (1) pathologically proven NPC, (2) 6 months or more interval from definitive chemoradiotherapy (if platinum was administered as definitive treatment), and (3) histology according to the WHO classification (I, squamous cell carcinoma; II, keratinizing undifferentiated carcinoma; III, non-keratinizing undifferentiated carcinoma). Exclusion criteria were as follows: (1) indication for definitive treatment (surgery or radiotherapy), (2) any prior systemic therapy for R/M NPC, and (3) history of allergy to any study treatment (PTX, CBDCA, and Cmab). This study was approved by the Institutional Review Board of the National Cancer Center East.

### Treatment

Chemotherapy consisted of PTX 100 mg/m^2^ on days 1 and 8; CBDCA area under the blood concentration–time curve (AUC) 2.5 on days 1 and 8, repeated every 3 weeks; and Cmab at an initial dose of 400 mg/m^2^, followed by 250 mg/m^2^ weekly, according to the CSPOR-HN02 study (11). PTX and CBDCA were administered for six cycles or until unacceptable toxicities occurred. Cmab was continued until disease progression or unacceptable toxicities. As a general principle, every antitumor drug was omitted if grade 3 or above toxicity occurred and then restarted after the toxicities were resolved. Doses of antitumor agents at the following cycle were modified when unacceptable toxicities defined as grade 4 or prolonged grade 3 toxicity was observed. Modification was performed by reducing the corresponding drug by 20% (e.g., if grade 4 neutropenia occurred, the dose of PTX and CBDCA was reduced from 100 to 80 mg/m^2^, and from AUC2.5 to 2.0, respectively, from the following treatment cycle) down to a minimum of 60% of the original dose. Toxicity during treatment was graded using the Common Toxicity Criteria for Adverse Event (CTCAE version 4.0).

### Evaluation of Efficacy and Statistical Analysis

Clinical response to treatment was evaluated radiographically using computerized tomography or magnetic resonance imaging approximately every 8 weeks until disease progression or treatment discontinuation. Antitumor activity was retrospectively evaluated by a single assessor according to the Response Evaluation Criteria in Solid Tumors (RECIST) v.1.1 (12) via the review of imaging results. After the completion of study treatment, disease progression, survival status, and any further anticancer treatment were documented until death or loss to follow-up. All disease progression was determined radiologically using computed tomography or magnetic resonance imaging. The event of PFS was defined as disease progression or death from any cause, while the event of OS was determined as death from any cause. PFS and OS were calculated by the Kaplan–Meier product-limit method. All other events were censored. All analyses were carried out using SPSS ver. 22 (IBM Corp., Armonk, NY, USA).

## Results

### Patients Characteristics

A total of 14 patients were included in the study. Patient characteristics are presented in Table 1.

**Table 1:** Patient characteristics.

**Characteristic (*****N*** **=14)**		**Patients, *n* (%)**
Sex	Male	10 (71.4)
	Female	4 (28.6)
Age (years)	Median	59.6
	Range	43-74
Performance status	0	9 (64.3)
	1	4 (28.6)
	2 or above	1 (7.1)
Histology, WHO classification	I	1 (7.1)
	II	4 (28.6)
	III	7(50.0)
	Unknown	2 (14.3)
EBV status^*^	Positive	3 (21.4)
	Negative	0 (0)
	Unknown	11 (78.6)
Staging at initial diagnosis	I	0 (0)
	II	1(7.1)
	III	5 (35.7)
	IV	8 (57.1)
Locoregional or distant metastasis	Locoregional only	5 (35.7)
	Locoregional and distant	8 (57.1)
	metastasis	
	Distant metastasis only	1 (7.1)
Recurrent/metastasis site^**^	Primary site	10
	Neck lymph node	8
	Lung	6
	Liver	4
	Bone	6
	Others	1
Treatment at initial diagnosis	CDDP+ RT	4 (28.6)
	TPF^***^+ RT	2 (14.3)
	Chemotherapy	8 (57.1)

* *Evaluated by EBV-encoded small RNAs (EBERs)–in situ hybridization. EBV, Epstein–Barr virus*.

** *Cumulative total number*.

*** *TPF: DTX+CDDP+5FU*.

### Efficacy

**Figure 1:**
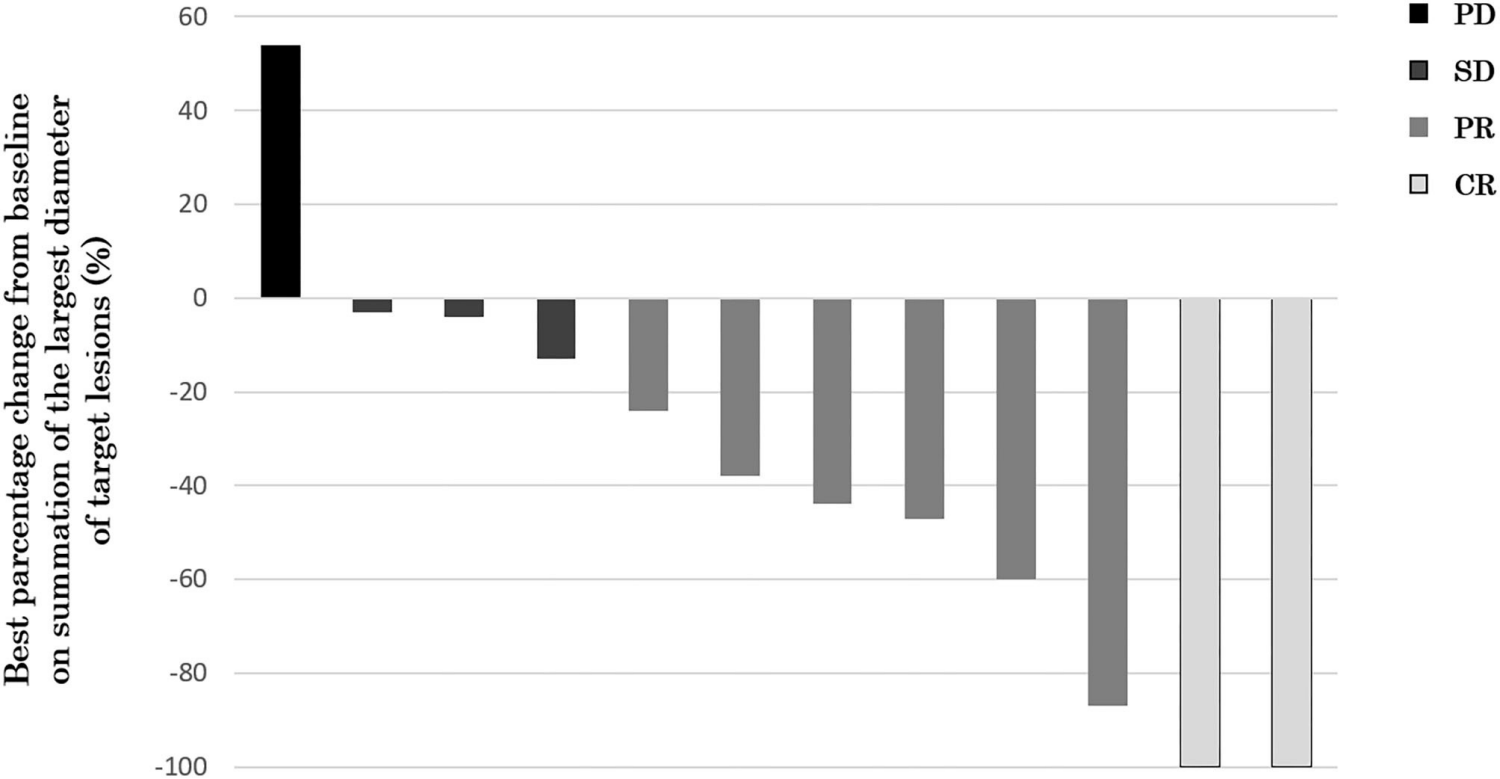
Waterfall plot of maximum percentage change from baseline on summation of the largest diameter of target lesions (*N* = 12). Two (16.7%) patients achieved a complete response, 5 (41.7%) achieved a partial response, and 4 (33.3%) were classified as stable disease, giving an ORR of 58.3% and a DCR of 91.7%.

**Figure 2:**
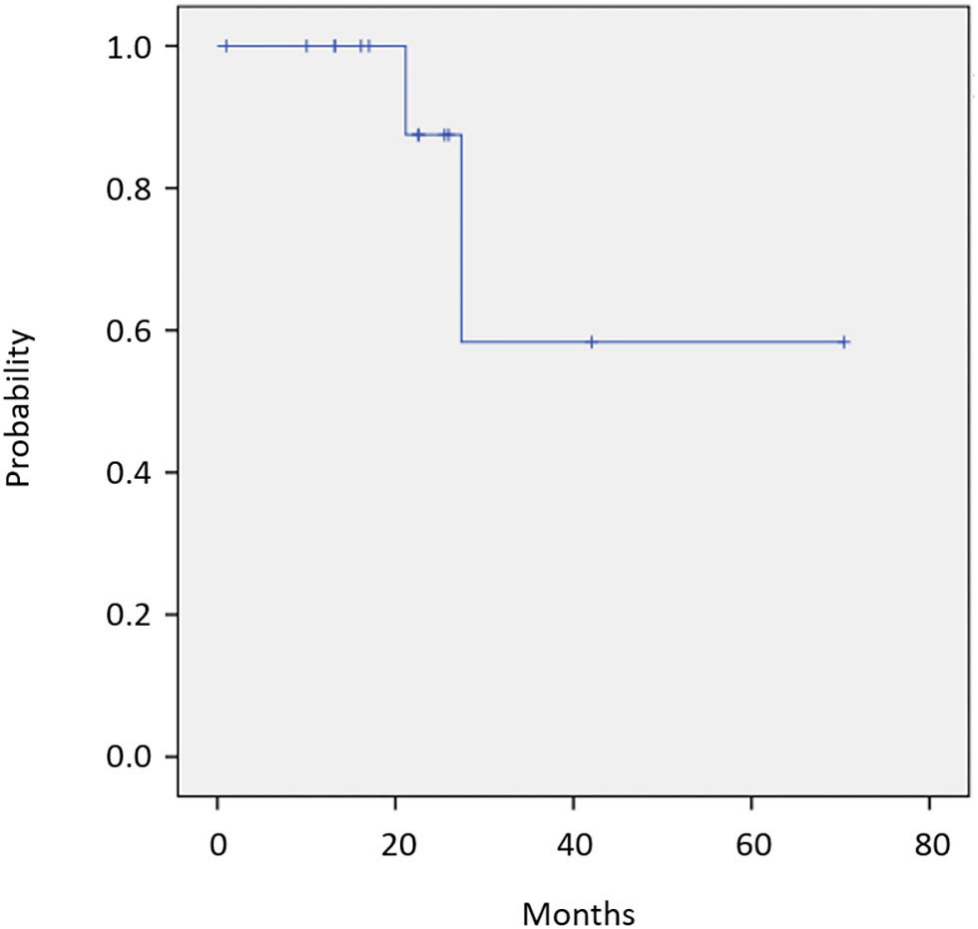
Overall survival (*N* = 14). OS was not reached with observed death events of 2.

**Figure 3:**
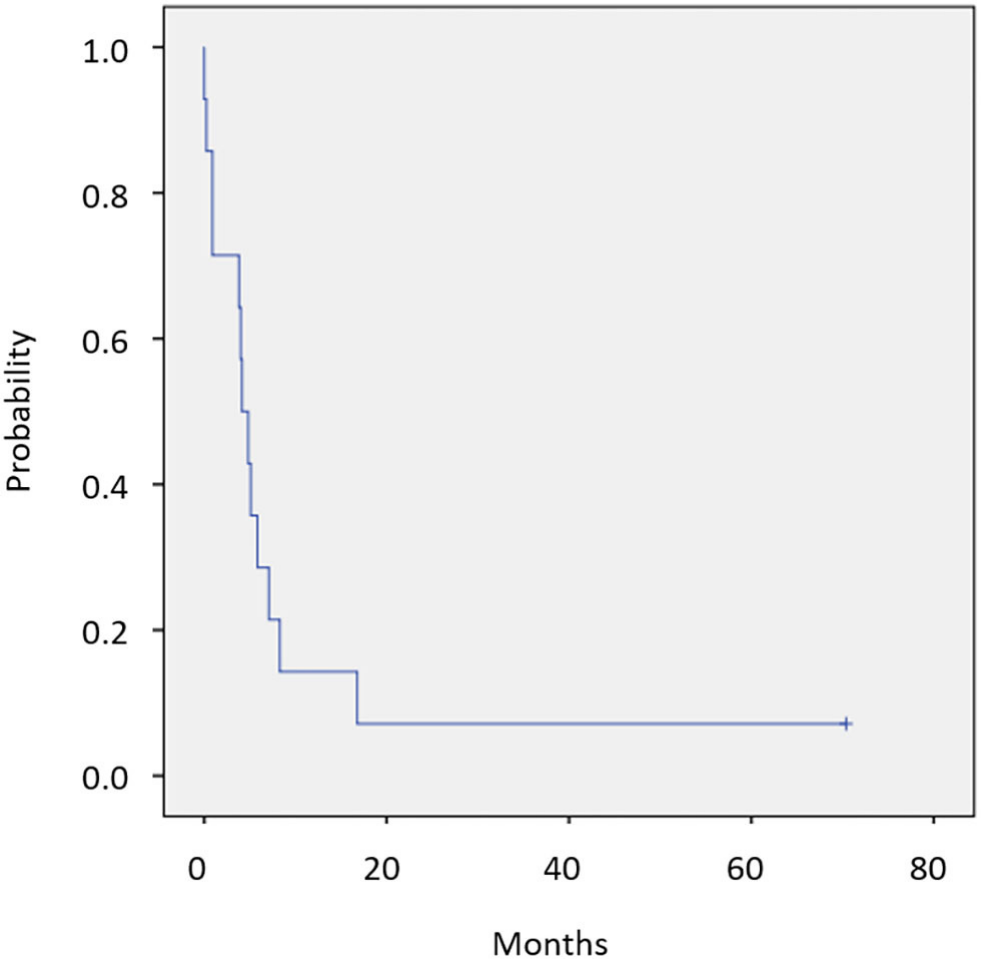
Progression-free survival (*N* = 14). Median PFS was 4.1 months (95% CI, 2.6–5.6 months).

Twelve of 14 patients were assessed for efficacy (Table 2, Figure 1). ORR was 58.3%. Two (16.7%) patients achieved a complete response, 5 (41.7%) achieved a partial response, and 4 (33.3%) were classified as stable disease, giving a DCR of 91.7%. With a median follow-up of 23.8 months, median OS was not reached with observed death events of 2 (Figure 2). Median PFS was 4.1 months [95% confidence interval (CI), 2.6–5.6 months] (Figure 3). Discontinuation of study treatment was due to disease progression (*n* = 10), adverse events (*n* = 2), and poor adherence to treatment (*n* = 1). Two patients experienced a mixed response (only part of the disease progressed while other parts maintained a response) during 4 and 6 months of Cmab maintenance. Both patients restarted PCE treatment and then achieved a partial response again. Of these two patients, one patient has been maintained for 26 months to date, with 6 months of PCE followed by PTX+Cmab (CBDCA was discontinued due to fatigue). The second patient maintained a partial response for 6 months, after which nivolumab was started due to disease progression.

**Table 2:** Best response by treatment.

**Characteristic (*N* = 12)**	**Patients, *n* (%)**
Complete response (CR)	2 (16.7)
Partial response (PR)	5 (41.7)
Stable disease (SD)	4 (33.3)
Progressive disease (PD)	1 (8.3)
Overall response rate (ORR)	7 (58.3)
Disease control rate	11 (91.7)

### Adverse Events

Adverse events are presented in Table 3. Grades 3–4 adverse events were neutropenia (21.4%) and skin rash (14.3%), and one case each of infusion reaction, anaphylactic shock caused by CBDCA (grade 4), bacterial pneumonia (grade 3), and ischemic colitis (grade 3). The grade 4 case of infusion reaction led to the discontinuation of PCE. Dose adjustment of PTX and CBDCA was required due to the following adverse events: four cases with decreased neutrophil count (one case of grade 4, two cases of grade 3, and one case of prolonged grade 2) and one case with nausea (grade 3). There was no treatment-related death.

**Table 3:** Adverse events.

**Adverse events (AE)**	**Any grade**	**Grade 3 or above**
	**patients, *n* (%)**	**patients, *n* (%)**
Skin rash	12 (85.7)	2 (14.3)
White blood cells decreased	10 (71.4)	4 (28.6)
Neutropenia	10 (71.4)	3 (21.4)
Anemia	10 (71.4)	0 (0.0)
Mucositis	7 (50.0)	0 (0.0)
Dry skin	6 (42.9)	0 (0.0)
Nausea/vomiting	6 (42.9)	1 (7.1)
Fatigue	5 (35.7)	0 (0.0)
Sensory disorder	5 (35.7)	0 (0.0)
Pruritus	4 (28.6)	0 (0.0)
Hypomagnesemia	4 (28.6)	0 (0.0)
Infusion reaction	3 (21.4)	1 (7.1)
Anaphylactic shock^*^	1 (7.1)	1 (7.1)
Diarrhea	3 (21.4)	0 (0.0)
Anorexia	3 (21.4)	0 (0.0)
Constipation	2 (14.3)	0 (0.0)
Platelet decreased	1 (7.1)	0 (0.0)
Trichomegaly	1 (7.1)	0 (0.0)
Interstitial pneumonitis	0 (0.0)	0 (0.0)
Any other AE^**^	7 (57.1)	2 (21.4)

* *CBDCA was suspected to be a cause of anaphylactic shock*.

** *Grade 3 or above AEs were bacterial pneumonia (grade 3) and ischemic colitis (grade 3)*.

### Subsequent Treatment

Details of subsequent treatment are shown in Table 4. A total of 10 (71.4%) patients received subsequent treatment after the discontinuation of study treatment. A patient who experienced a grade 4 infusion reaction received CBDCA+PTX as second-line treatment. A total of six patients received nivolumab as subsequent treatment, five patients received nivolumab as second-line treatment, and one received nivolumab after progression in PCE and following CDDP+RT for local disease. Three of five patients who received nivolumab as a second-line treatment achieved a partial response. The nivolumab cohort showed a trend toward better OS with no OS event compared with the others without nivolumab (median OS; not reached vs. 27.4 months) [(95% CI, 21.2–not reached), *p* = 0.317]. Two patients received re-administration of PCE after Cmab maintenance therapy due to disease progression, both of whom responded well.

**Table 4:** Subsequent treatment.

**Subsequent treatments**		
**Line**
	No subsequent treatment (PCE only)	2
	Until 2nd line	6
	Until 3rd line	2
	Until 4th line or above	2
Regimen of subsequent treatments after PCE^*^^a^		
	Nivolumab	6
	S-1	4
	CBDCA+PTX^*^^b^	1
	PTX^*^^c^	1
	DTX	1
	GEM^^*^d^	4
	CDDP+RT for local disease^*^^e^	1
**Number of responded patients in 2nd-line treatment**, ***N*** **(CR or PR/total** ***N*****)**		
	Nivolumab^*^^f^	3/5
	S-1	1/3
	PTX	1/1

* a Cumulative total. CBDCA: carboplatin, PTX: paclitaxel, DTX: docetaxel, GEM: gemcitabine, CDDP: cisplatin, RT radiotherapy. *b One patient experienced an infusion reaction to cetuximab in PCE, and the regimen was changed to CBDCA+PTX. *c One patient experienced an allergy to CBDCA in PCE, and the regimen was changed to cetuximab alone. Disease progressed after 3 months of cetuximab maintenance, then PTX alone was started and PR was achieved. *d Off-label use in Japan. GEM use was reviewed and approved by our institutional review board. *e Disease progressed after 4 months of CDDP+RT, then nivolumab was started. *f Two PD cases treated with nivolumab showed a mixed response (part of the disease progressed while other parts maintained a response) and received palliative radiotherapy, then maintained PR in the other disease. One PR case could not be evaluated by RECIST and showed a response in a non-measurable lesion.

## Discussion

In this study, the first report of PCE therapy as first-line treatment for R/M NPC to date, the results showed manageable toxicity and promising activity for this regimen, with an ORR of 58.3% and not reaching median OS on a median follow-up of 23.8 months in first-line treatment for R/M NPC. Toxicities were manageable and were tolerated in the outpatient clinic.

The results of this study and other reports on treatment for R/M NPC are shown in Table 5. GEM+CDDP showed a good survival benefit, with an ORR of 64% and a median OS of 29.1 months on a median follow-up of 22.0 months in the same setting (2). On the other hand, GEM is not covered by insurance in some countries, including Japan; in these countries, an EGFR inhibitor-containing regimen is sometimes used as first-line treatment for R/M NPC. CDDP+5FU+Cmab (EXTREME regimen) is considered an option but may affect patient QoL because of the severe nausea caused by CDDP and the need for deep vein catheterization for 5FU, which, in many cases, requires inpatient care. In this situation, PCE may be a better treatment option as first-line treatment, considering its encouraging efficacy and acceptable toxicity profile compared to CDDP-based regimens. Moreover, PCE can also be used as an option after progression during GEM maintenance therapy following GEM+CDDP, since no standard therapy has been established in this situation.

**Table 5:** Palliative chemotherapy for R/M NPC.

**Author**	**Phase**	**Line**	**Treatment**	**RR (%)**	**Median OS (months)**	**Median PFS (months)**
Zhang et al. (2)	III	First	GEM+CDDP	64	29.1	7
			5FU+CDDP	42	20.9	5.6
Chan et al. (9)	II	Platinum-refractory	Cmab+CBDCA	11.7	7.8	2.7
Xu et al. (13)	Retrospective	First	Cmab included^*^	70	23.6	12.2
Present study	Retrospective	First	PTX+CBDCA+Cmab	58.3	NA^*^	4.5

* *NA, not available*.

*NPC, nasopharyngeal cancer; GEM, gemcitabine; CDDP, cisplatin; Cmab, cetuximab; CBDCA, carboplatin; PTX, paclitaxel*.

*(13) is cited only in Table 5.

The drugs used in the PCE regimen collaboratively facilitate tumor apoptosis using different pathways. For example, Cmab promotes cell cycle arrest and activation of proapoptotic molecules through the inhibition of EGFR signaling, taxanes inhibit microtubule disassembly, and platinum agents form DNA adducts (14–16). GEM and platinum combination is also reported to have synergetic cytotoxicity via the inhibition of DNA synthesis and increasing cell apoptosis (17). On the other hand, Cmab, part of the PCE regimen, is well-known to have immunomodulatory effects represented by antibody-dependent cell-mediated cytotoxicity (ADCC) upon cross-linkage between Natural Killer (NK) cells and tumor cells, besides its direct effects on tumor cells (18). Notably, the combination of Cmab and taxane has shown additive NK cell-mediated antitumor immunological ability (19). Given the reduced cytotoxic activity of NK cells in NPC and an inverse association between the degree of intratumoral NK cell infiltration and prognosis, the present encouraging clinical activity of the PCE regimen might be considered to be based on an augmented NK cell-driven antitumor immunity (20–22). Besides these interaction mechanisms, Cmab maintenance therapy may also contribute to the prolongation of PFS and OS. In a phase 3 study of GEM+CDDP for R/M NPC, GEM was continued as maintenance therapy after six cycles of CDDP, while 5FU+CDDP finished after a maximum of six cycles (2). This maintenance therapy may have had some effect on the superior prognosis of GEM+CDDP against 5FU+CDDP. Similarly, Cmab maintenance therapy has been widely used for R/M HNSCC, and its safety and feasibility have been reported (23). Although not all patients might benefit from Cmab maintenance, some patients achieved long-term disease control with maintenance Cmab therapy after six cycles of PCE.

In this study, while neutropenia of any grade was observed in 71.4%, no febrile neutropenia developed. Likewise, in a phase 2 study of PCE for HNSCC, grade 3 or 4 neutropenia was observed in 68%, while only 9% of patients developed grade 3 febrile neutropenia, allowing for safe outpatient management (11). Moreover, the main adverse events of PCE were hematologic events, whereas subjective symptoms such as nausea and appetite loss were less common than with other regimens. The skin adverse events were frequent but manageable with appropriate skin care. In fact, grade 3 or above skin adverse events occurred in only 14.3% of patients, suggesting that PCE ensures a good quality of life. Despite the higher age of enrolled patients in the current study compared to GEM+CDDP (median 59.6 vs. 47 years), which potentially correlates with severe toxicity (24, 25), the toxicity profile of PCE is considered equivalent to or partially more favorable than that of the historical GEM+CDDP cohort (e.g., current study vs. GEM+CDDP in grade 3 or above AE: neutropenia, 21.4 vs. 21%; anemia, 0 vs. 3%; thrombocytopenia, 0 vs. 11%) (2). Accumulating evidence has shown better tolerability with similar efficacy of CBDCA over CDDP in NPC (26, 27). Furthermore, because the regimen was administered weekly, the chemotherapy dose can be adjusted immediately when adverse events arise. Besides, the weekly administration itself might also contribute to the favorable toxicity profile. A randomized phase III study for breast cancer reported that the lower weekly dose of PTX arm showed lesser toxicity despite a higher dose intensity (mg/m^2^/week) than the once in 3 weeks high-dose PTX arm (28).

Re-administration of PCE can be considered if a mixed response (partial shrinkage and partial progression of disease) is observed during Cmab maintenance therapy. In this study, PCE was restarted in two patients and achieved a good response in both cases. Accumulated toxicity such as sensory neuropathy or severe cytopenia is sometimes relieved after a period of drug withdrawal. CBDCA allergy should be considered in cases with multiple dose administration, but most severe adverse events will be avoided with a drug holiday. Re-administration of PCE, or PTX+Cmab in those with CBDCA allergy, can be a promising strategy in patients who achieve a response during the first PCE administration.

Immune checkpoint inhibitor (ICI) therapy after PCE may have prolonged survival in this study. Three of five patients who received second-line nivolumab after PCE showed a favorable response, which tends to better than that reported previously (29, 30). Further, although the nivolumab cohort tended to have a shorter follow-up period, patients who received nivolumab showed a trend toward better OS compared to those who did not. Nivolumab, an anti-PD-1 antibody, has been approved in platinum-refractory R/M HNSCC. Although data on the efficacy of nivolumab in R/M NPC are limited, promising efficacy has been reported, with RRs of 12.5–37.5% (29, 30). Several studies have reported that certain chemotherapies may enhance responses to ICIs. In particular, taxanes such as paclitaxel showed an interaction potential with immunotherapy via the activation of toll-like receptor activity or dendritic-cell activity (31, 32). In a phase 3 clinical trial of breast cancer, combination therapy of nab-paclitaxel and the anti PD-L1 inhibitor atezolizumab showed prolonged PFS over nab-paclitaxel alone in the PD-L1-positive subgroup (33). Besides, in the exploratory analysis of 2nd-line therapy after either TPExtreme regimen, which was combination therapy of docetaxel+CDDP+Cmab, or ETREME regimen in R/M HNSCC, patients treated with ICI showed longer OS compared with those with chemotherapy in both regimens; however, this trend was more potent in TPExtreme (interaction test *p* = 0.077) (34). This result also suggested that the use of taxane prior to ICI presumably augments the effect of subsequent ICI and supports our hypothesis that PTX may enhance the response to nivolumab. On the other hand, in a multicenter retrospective study of R/M HNSCC, a taxane-based regimen after ICI was associated with higher ORR than other chemotherapy regimens (53 vs. 25%, *p* = 0.024) (35). Also, according to a retrospective study of metastatic melanoma, an increased proportion of CD8-positive cells with elevated PD-1 and CD69 expression was observed while on chemotherapy as compared with all-time points on ICIs, suggesting immune-activation by interaction of chemotherapy and ICIs (36). For R/M HNSCC, pembrolizumab or pembrolizumab+platinum+5FU showed significantly better OS than the EXTREME regimen and became a standard first-line treatment (37). These treatments have also been available for use in R/M NPC in Japan from December 2019. As to future prospects, PCE therapy may enhance therapeutic outcomes after first-line pembrolizumab +/– chemotherapy by an interaction between immunotherapy and PTX. The best treatment sequence when both an ICI and chemotherapy can be used remains unknown, and prospective evaluation is also warranted.

Several limitations of our study warrant mention. First, it was conducted under a retrospective design with a limited number of patients and no control arm. The paucity of studies on Cmab for R/M NPC made it difficult to establish a historical control for comparison. Although we believe that it is worth noting that our results indicate a promising tumor response equivalent to those with standard therapies, such as GEM+CDDP and 5FU+CDDP, together with a favorable toxicity profile, prospective confirmation in a large number of patients is necessary. Second, assessment of expression pattern for EGFR and Epstein–Barr Virus (EBV) was not routinely performed in participating cases. Regarding EGFR, pathological EGFR expression rate is 73.3–84.1% with NPC in general (5, 38–40), and 85% with R/M NPC (9), and high expression of EGFR is correlated with an unfavorable prognosis in NPC (4, 5). Cmab has been used for R/M HNSCC regardless of EGFR expression, based on a sub-analysis of the EXTREME study in which EGFR copy number was reported to be not predictive of Cmab efficacy (41). EGFR expression was detected in 98% of cases of HNSCC (3), which supports the use of anti-EGFR targeted therapy for HNSCC without individual EGFR testing. EGFR expression in NPC is reported to be about 80% (5), which is nearly as high as in other HNSCCs. Considering this background, Cmab can possibly be considered regardless of EGFR expression in NPC, as with HNSCCs. As for EBV, this infects more than 95% of the world population and is associated with multiple malignancies, including NPC. WHO-II and -III tumors, in which the EBV-positive rate is almost 100%, and which account for 95% of NPC cases in China and Southeast Asia, and up to 75% in Japan and North America (42–44), are considered to be primarily caused by EBV-driven carcinogenesis. Latent membrane protein 1 of EBV, a viral oncogene that plays an important role in the carcinogenesis of NPC, is reported to induce EGFR expression (38, 45). These markers may be suitable biomarkers for the efficacy of PCE in this population. Third, prognosis may differ depending on treatment period due to a difference in subsequent therapies. In Japan, nivolumab was approved for all types of head and neck cancer in 2017. Consequently, most patients received nivolumab as a second-line therapy after 2017, while those before this time did not receive immunotherapy. Since nivolumab showed promising activity in our study, prognosis would differ depending on accessibility to immunotherapy, and this factor warrants further investigation.

## Conclusion

Although the number of study population was small, our results suggest that PCE is feasible and potentially effective for R/M NPC, with a 58.3% response rate and 4.1-month PFS. Further prospective evaluation is warranted.

## Data Availability Statement

All datasets generated for this study are included in the article/supplementary material.

## Ethics Statement

The studies involving human participants were reviewed and approved by The Institutional Review Board of the National Cancer Center East. Written informed consent for participation was not required for this study in accordance with the national legislation and the institutional requirements. Written informed consent was not obtained from the individual(s) for the publication of any potentially identifiable images or data included in this article.

## Author Contributions

YU and MT conceived of the presented idea. YU and TF developed the theory and performed the computations. TF and KI verified the analytical methods. TE, SO, and MT supervised the findings of this work. All authors discussed the results and contributed to the final manuscript. All authors contributed to the article and approved the submitted version.

## Conflict of Interest

The authors declare that the research was conducted in the absence of any commercial or financial relationships that could be construed as a potential conflict of interest.
